# Machine learning models in post-stroke aphasia: a scoping review

**DOI:** 10.3389/fneur.2026.1806856

**Published:** 2026-05-07

**Authors:** Xiaoxue Li, Hengjie Song, Ningjing Guo, Congmin Kang, Xiaoyan Gong, Xinyu Ji, Jie Zheng

**Affiliations:** 1 School of Nursing, Shanxi Medical University, Shanxi, China; 2 The Fifth People’s Hospital of Shanxi Province, Shanxi, China

**Keywords:** aphasia, machine learning, nursing care, scoping review, stroke

## Abstract

**Objective:**

To systematically review the literature on the application of machine learning models in post-stroke aphasia, and to provide a reference for the construction and clinical application of related models.

**Methods:**

Based on scoping review methodology, we searched Web of Science, PubMed, Cochrane Library, Embase, CINAHL, CNKI, VIP database, Wanfang database, and China Biology Medicine. The search time limit was from the database’s establishment to November 20, 2025, and the retrieved literature was screened, summarized, extracted, and analyzed.

**Results:**

A total of 19 articles were included. The analysis results showed that the machine learning algorithms used in post-stroke aphasia models were mainly supervised methods, including random forests, neural networks, and support vector machines. The data sources of the model were diverse. The indicators included in the model covered multimodal data. The functions of the model include diagnosis and classification of aphasia patients, assessment and prediction of the severity of aphasia patients, prediction of the language function and rehabilitation outcome of patients, monitoring and evaluation of symptoms, etc.

**Conclusion:**

Machine learning models have high applicability and broad scope in post-stroke aphasia. Future research still requires multi-center, multi-modal data and external validation to enhance its robustness and clinical feasibility.

## 1 Introduction

Stroke is one of the leading causes of death and disability worldwide, which brings a heavy disease burden to patients and their families (1). Stroke patients often experience multiple functional disorders, including motor, sensory, swallowing, and language issues (2, 3). Among these, aphasia is an acquired communication disorder characterized by difficulty with language. It results from varying degrees of damage to the language centers of the brain (usually located in the left hemisphere), affecting oral expression, reading ability, writing ability, language comprehension, and even cognitive and computational functions (4, 5). Studies have shown that approximately 30% of stroke patients will have secondary aphasia, with the global incidence of new cases reaching up to 4.5 million annually (5–7). Of these, 30 to 43% still have language dysfunction beyond 6 months after stroke (8, 9). Aphasia not only seriously affects the daily life function of patients, but also reduces their quality of life, and may even cause psychological problems such as depression (10, 11).

At present, the clinical practice of aphasia still faces several challenges, including the long time consumption and insufficient consistency of results of traditional assessment tools (12), as well as the scarcity of treatment resources (13), which complicates the delivery of intensive and personalized rehabilitation. With the development of artificial intelligence, a large amount of complex data in aphasia can be deeply mined, and predictive models can be constructed through machine learning to realize automatic assessment and personalized rehabilitation, which provides new possibilities for accurate identification, efficacy prediction, and rehabilitation intervention of aphasia (14, 15). While studies on the application of machine learning in aphasia have been conducted, they are predominantly concentrated in foreign countries, with relatively few studies available in China. Although there have been studies reporting the application of machine learning in post-stroke aphasia, the existing reviews mostly focus on a single algorithm or a single data type, lacking a systematic comprehensive analysis of different task types and data modalities. Therefore, guided by the scoping review methodology (16), this study systematically searched relevant databases both domestically and internationally, used the Joanna Briggs Institute (JBI) quality assessment tool to evaluate the methodological quality of the included literature, systematically analyzed the methodological characteristics of existing studies from dimensions such as sample size, validation methods, and data sources, and summarized the patterns of model performance across different tasks and data modalities. Compared with the existing literature, this study not only summarizes the current research status, but also extracts the reference and guidance significance for future research design, model optimization and clinical application, highlighting its unique contribution and application value in this field.

## 2 Materials and methods

This scoping review was conducted in accordance with the scoping review methodology and reported following the Preferred Reporting Items for Systematic Reviews and Meta-Analyses extension for Scoping Reviews (PRISMA-ScR) guidelines for literature search, screening, data extraction, and result reporting to enhance the transparency and reproducibility of the review.

### 2.1 Determine the research question

(1) What are the main types of machine learning algorithms used in machine learning models of post-stroke aphasia? (2) What are the data sources and model inclusion indicators for machine learning models of post-stroke aphasia? (3) What are the functions of machine learning models of post-stroke aphasia, and what is the performance regarding model fitting or application?

### 2.2 Search strategy

We systematically searched Web of Science, PubMed, Cochrane Library, Embase, CINAHL, China National Knowledge Infrastructure, VIP database, Wanfang Database, and China Biology Medicine Disc by combining subject terms, free words, and Boolean logic operators. The search time limit was from the database’s establishment to November 20, 2025. Chinese databases take CNKI as an example, The retrieval formula is SU = (machine learning + artificial intelligence + supervised learning + unsupervised learning + reinforcement learning + decision tree + neural network + support vector machine + Bayesian classifier + clustering + dimensionality reduction + metric learning + K-nearest neighbor classification + random forest + ensemble learning + deep learning + logistic regression + natural language processing) AND SU = (stroke + cerebrovascular accident + cerebral hemorrhage + post-stroke + subarachnoid hemorrhage + cerebral infarction + cerebral ischemia + cerebral embolism) AND SU = (aphasia + language disorder + speech disorder + communication difficulty + communication disorder). The English search format takes PubMed as an example, and the search strategy is shown in Table 1.

**Table 1:** PubMed search strategy.

Steps	PubMed
#1	“Stroke”[Mesh]
#2	“Cerebrovascular Accident”[Title/Abstract] OR “CVA”[Title/Abstract] OR “poststroke”[Title/Abstract] OR “post-stroke”[Title/Abstract] OR “cerebral hemorrhage”[Title/Abstract] OR “subarachnoid hemorrhage”[Title/Abstract] OR “cerebral infarction”[Title/Abstract] OR “brain infarction”[Title/Abstract] OR “cerebral ischemia”[Title/Abstract] OR “ischemic stroke”[Title/Abstract] OR “cerebral embolism”[Title/Abstract]
#3	#1 OR #2
#4	“Aphasia”[Mesh]
#5	“dysphasia”[Title/Abstract] OR “language disorders”[Title/Abstract] OR “speech disorders”[Title/Abstract] OR “communication difficulties”[Title/Abstract] OR “communication disorders”[Title/Abstract]
#6	#4 OR #5
#7	“Machine Learning”[Mesh]
#8	“supervised learning”[Title/Abstract] OR “unsupervised learning”[Title/Abstract] OR “intensive learning”[Title/Abstract] OR “deep learning”[Title/Abstract] OR “decision tree”[Title/Abstract] OR “support vector machine”[Title/Abstract] OR “Bayesian classifier”[Title/Abstract] OR “Bayesian network”[Title/Abstract] OR “ensemble learning”[Title/Abstract] OR “multiclassifier”[All Fields] OR “multiclassifiers”[All Fields] OR “learning”[Title/Abstract] OR “committee-based”[All Fields] OR “clustering”[Title/Abstract] OR “dimensionality reduction”[Title/Abstract] OR “metric learning”[Title/Abstract] OR “k-nearest”[All Fields] OR “neighbor learning”[Title/Abstract] OR “neural networks”[Title/Abstract] OR “random forest”[Title/Abstract]
#9	#7 OR #8
#10	#3 AND #6 AND #9

### 2.3 Literature screening

The literature screening was conducted in two steps: Firstly, the retrieved literature was imported into End Note 21 software. After removing duplicate literature, two researchers independently screened the titles and abstracts to exclude obviously irrelevant literature; Secondly, the remaining literature was read in full to determine whether it met the inclusion criteria. The literature inclusion criteria were determined according to the principles of PCC (17): (1) Participants (P) were diagnosed with post-stroke aphasia. (2) Concept (C) refers to machine learning algorithms, including supervised learning, unsupervised learning, reinforcement learning, deep learning, etc. (3) Context (C) is in the medical and health field, particularly in the scenarios related to stroke rehabilitation and care; the research type is original research, including quantitative research, qualitative research, and mixed-methods research. Exclusion criteria: (1) Abstracts of conferences, without access to full texts; (2) Content identical or duplicated publications; (3) Non-Chinese or non-English literature.

### 2.4 Data extraction and synthesis

Both researchers independently extracted and summarized data using Excel, including author, publication year, country, research type, study subjects, sample size, and model function. During literature screening, data extraction, and summary, any disagreements were discussed until consensus was reached or a third researcher was consulted for judgment.

### 2.5 Literature quality assessment

The quality of the included literature was evaluated using the literature quality assessment items from the JBI Evidence-Based Healthcare Center in Australia (18). According to the research design of each article, such as randomized controlled trials, cohort studies, quasi-experimental studies, cross sectional studies, etc., the corresponding assessment items were selected. The quality evaluation was independently conducted by two researchers. In case of disagreement, a third researcher was consulted to resolve the disagreement. The quality evaluation results were presented in Table 2 in the form of the number of “yes” items divided by the total number of items.

**Table 2:** Basic characteristics of the included literature.

Author (year)	Country	Study Subjects/ Sample Size	Model function	Validation method	Model inclusion indicators	Model performance	Literature quality assessment (number of “yes” entries/total number of entries)
Zhong et al. (2025) (19)	China	Patients with stroke /212	Patients with post-stroke aphasia were distinguished from those without aphasia	Internal validation (80% training set, 20% test set), external validation	Neuroimaging data (Resting-state fMRI brain functional network topology metrics)	Accuracy: 88.70%, Precision: 87.34%, Recall: 99.32%, Specificity: 58.00%, F1-score: 92.92%, AUC: 0.92	6/9
Marte et al. (2025) (20)	United States	Spanish-English bilingual patients with post-stroke aphasia /48	Predicting patient response after naming therapy	Internal Validation	General demographic data (years of education, age, etc.); clinical indicators (severity of language aphasia treatment, non-verbal cognitive performance, bilingual experience, etc.)	F1-score: 0.767–0.790, accuracy: 78.3–85%, precision: 80–80.8%, recall: 81–81.9%, specificity: 74.7–88.4%.	7/13
Hu et al. (2025)(21)	United States	Patients with aphasia after chronic stroke /76	Predict the severity of the patient’s aphasia	Internal Validation	General demographic data (age, years of education, number of months after stroke, etc.); neuroimaging data (lesion volume, percentage of gray matter in the left hemisphere, resting-state functional magnetic resonance imaging, etc.)	–	6/8
Metu et al. (2024) (22)	United States	Patients with post-stroke aphasia	Identifying the types of language fluency in patients with aphasia	Internal Validation, external Validation	Physiological signal data (audio spectral graph, acoustic features, speech-to-text sequences)	Convolutional Neural Network (CNN) accuracy: 81%; Recurrent Neural Network (RNN) accuracy: 83%	6/8
Riccardi et al. (2024) (23)	United States	Patients with chronic stroke /71	Predict the patient’s aphasia quotient	Internal Validation	Clinical indicators (lexical diversity, average sentence length, etc.); neuroimaging data (proportion of damage in 64 brain regions)	–	6/8
Teghipco et al. (2024) (24)	United States	Patient with chronic stroke /231	Predict the severity of the patient’s aphasia	Internal Validation	Neuroimaging data (brain structural imaging data, lesion areas, morphometric diagrams, etc.)	F1-score: 0.70, Accuracy: 0.77, Precision: 0.59	6/8
Močilnik et al. (2024) (25)	Slovenia	Stroke patient with Broca’s aphasia /8	Distinguish whether the patient is in the pre-recovery stage or the post-recovery stage	Internal Validation	Physiological signal data (electroencephalogram functional connectivity indicators)	Accuracy: 89.4%, Precision: 0.95, Sensitivity: 0.82, Specificity: 0.96, F1-score: 0.88	8/9
Hildesheim et al. (2024) (26)	Canada	Patients with post-stroke aphasia /76	Predicting the language function of patients after stroke	Internal Validation	General demographic data (age, gender, years of education, etc.); clinical indicators (initial language score, non-verbal cognitive ability, disease duration, etc.); neuroimaging data (lesion volume, etc.)	–	8/9
Mahmoud et al. (2023) (27)	China	Patients with aphasia /12; Healthy subjects /34	Identification and classification of the speech of patients in naming and repetition tasks	Internal Validation	Physiological signal data (voice signal converted into high-resolution time-frequency images)	Accuracy: 59.17%	5/8
Jeong et al. (2022) (28)	Korea	Patients with aphasia after acute stroke /176	Predict the patient’s aphasia quotient	Internal Validation	General demographic data (age, gender, years of education); clinical indicators (magnetic resonance imaging examination and time interval from onset); neuroimaging data (fractional volume of diffusion-weighted imaging lesions, total volume of lesions)	Accuracy: 61.00%	6/8
Billot et al. (2022) (29)	United States	Patients with aphasia after chronic stroke /55	Predict the therapeutic responsiveness of patients after treatment	Internal Validation	General demographic data (age, educational level, post-stroke time); clinical indicators (aphasia quotient, cognitive composite score); neuroimaging data (lesion volume, proportion of gray matter/white matter preservation, etc.)	F1-score: 0.94, Accuracy: 92.7%, Precision: 91.4%, Recall: 97.0%	7/9
Landrigan et al. (2021) (30)	United States	Patients with post-stroke aphasia /226	Data-driven classification of aphasia subtypes and prediction of lesion sites	Internal Validation	Clinical indicators (20 psycholinguistic tests); neuroimaging data (binary damage status of 150 brain regions)	Accuracy: 76.9%	6/8
Mahmoud et al. (2020) (31)	China	Patients with aphasia /12; Healthy subjects /34	Evaluate the clarity of the patient’s speech	Internal Validation	Physiological signal data (high-resolution time-frequency images)	–	6/8
Kristinsson et al. (2020) (32)	United States	Patients with aphasia after chronic stroke /116	Predict the overall severity of the patient’s language disorder	Internal Validation	General demographic data (gender, age, time after stroke); clinical indicators (scores of the Western Aphasia Battery); neuroimaging data (brain blood flow, etc.)	–	8/8
Wilmskoetter et al. (2019) (33)	United States	Patients with aphasia after chronic stroke /5	Predict the patient’s response in the picture naming task	Internal Validation	Physiological signal data (high-density electroencephalogram signals)	Accuracy: 63–80%, AUC: 0.62–0.75, Sensitivity: 0.63–0.78, Specificity: 0.63–0.73	6/8
Blom-Smink et al. (2019) (34)	Netherlands	Patients with aphasia after moderate to severe stroke /88 cases	Predicting patients’ communication abilities following inpatient rehabilitation	Internal Validation	Clinical indicators (daily verbal communication ability score, speech processing ability)	AUC: 0.91	7/8
Gillespie et al. (2018) (35)	United States	Patients with aphasia after stroke /20	Identify the changes in the emotional state of the patient during the conversation	Internal Validation	Clinical indicators (self-assessed emotional state labels); physiological signal data (rhythm features, spectral features, glottal features, etc.)	–	5/8
Pustina et al. (2017) (36)	United States	Patients with aphasia after chronic stroke /53	Predict the severity of the patient	Internal Validation	Neuroimaging data (lesion maps, resting-state functional magnetic resonance imaging, lesion volume, etc.)	–	6/8
Yourganov et al. (2015) (37)	United States	Patients with aphasia after chronic stroke /98	Predicting the type of aphasia in patients	Internal Validation	Neuroimaging data (proportion of brain region damage)	Accuracy: 93.56%	6/8

AUC, Area under the Curve.

## 3 Results

### 3.1 Literature search results

**Figure 1:**
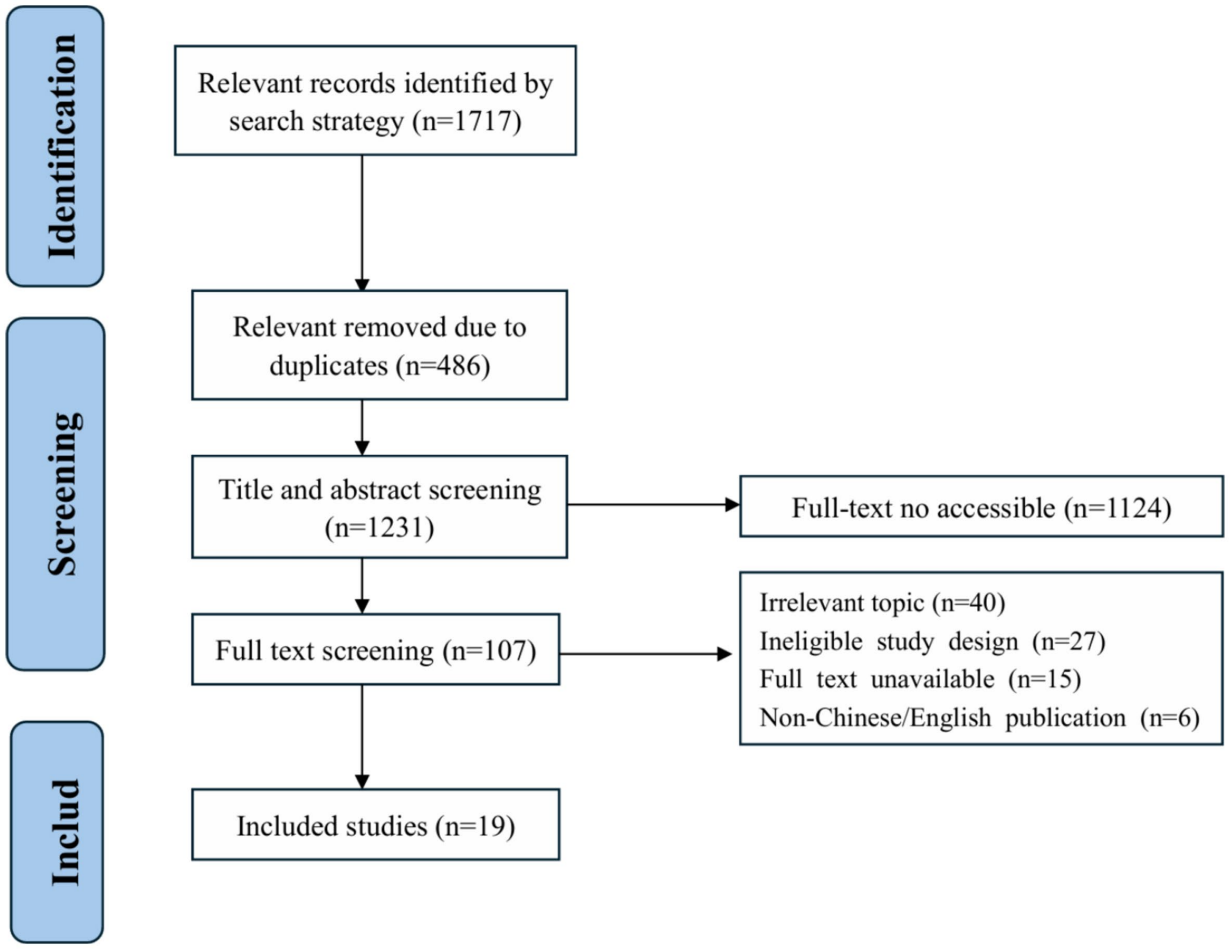
PRISMA flow chart of the selection process.

The initial search yielded 1717 documents; after removing duplicates, 1,231 remained. After screening of titles and abstracts, 107 documents were retained for full-text review. After reading the full texts and tracing the references, 19 documents were included (19–37). The flowchart of literature screening is shown in Figure 1.

### 3.2 Basic characteristics of included literature

The 19 included literature were published between 2015 and 2025, coming from 6 countries, including the United States (20–24, 29, 30, 32, 33, 35–37) (*n* = 12), China (19, 27, 31) (*n* = 3), South Korea (28) (*n* = 1), Slovenia (25)(*n* = 1), Canada (26) (*n* = 1), and the Netherlands (34) (*n* = 1). The study subjects mainly focused on patients with post-stroke aphasia, especially those in the chronic phase. The research designs included seven prospective studies (20, 25, 27, 29, 31, 34, 35), 11 retrospective studies (19, 21, 23, 24, 26, 28, 30, 32, 33, 36, 37), and 1 mixed-methods study (22). The basic characteristics of the included literature are presented in Table 2.

### 3.3 Types of machine learning algorithms applied in machine learning models of post-stroke aphasia

Among the included studies, 18 (19–29, 31–37) employed supervised machine learning algorithms, including support vector machines, random forests, decision trees, logistic regression, and various neural networks (such as feedforward neural networks, convolutional neural networks, and recurrent neural networks). One study (30) used unsupervised machine learning algorithms, including clustering. Seven studies (19–21, 24, 27, 29, 31) used multiple algorithms to construct models and compare them.

### 3.4 Data sources and model inclusion indicators of machine learning models for post-stroke aphasia

#### 3.4.1 Data sources

All 19 included studies reported the sources of data, which were primarily derived from the study participants themselves. (1) Data from patients, including general demographic data, clinical indicators, neuroimaging data, and physiological signal data. (2) Some studies (22, 27, 31) also included speech or behavioral data of healthy subjects as a control or training basis. (3) In addition, one study (22) utilized video data from public video platforms, and another study (19) used data from public image databases.

#### 3.4.2 Indicators included in the model

The indicators included in the model included (1) General demographic data, including age, gender, years of education, etc. (2) Clinical indicators, including cognitive score, aphasia quotient, daily verbal communication ability score, etc. (3) Neuroimaging data, including lesion volume, location and brain functional network topology metrics, etc. (4) Physiological signal data, including electroencephalogram functional connectivity measures, speech acoustic features, etc.

### 3.5 Evaluation metrics of the machine learning models for post-stroke aphasia

The evaluation metrics used to assess model performance in the included studies were varied. Twelve studies (19, 20, 22, 24, 25, 27–30, 33, 34, 37) mainly used accuracy, AUC, F1-score, precision, recall, and other indicators. Seven studies (21, 23, 26, 31, 32, 35, 36) primarily relied on error metrics, such as root mean square error and mean absolute error, combined with indicators, such as the Pearson correlation coefficient and coefficient of determination, to provide a more comprehensive assessment.

### 3.6 Functions and effects of machine learning models for post-stroke aphasia

#### 3.6.1 Diagnosis and classification of aphasia patients

Four studies (19, 22, 30, 37) constructed diagnostic and classification models of aphasia by evaluating general demographic data, neuroimaging data, and clinical indicators of patients, but there were differences in model function, fitting, and application effect. In terms of model function, three models (19, 30, 37) focus on subtype classification or diagnosis of aphasia according to brain damage patterns or clinical indicators. For example, the model constructed by Yourganov et al. (37) can automatically identify classical types such as Broca’s aphasia and Wernicke’s aphasia according to the proportion of brain damage. One model (22) uses speech acoustic features to automatically identify the fluency type of aphasia. In terms of the model performance, Yourganov et al. (37) reported an accuracy rate of 93.56%, and Zhong et al. (19) reported an area under the receiver operating curve of 0.92, indicating a good prediction performance of the model. However, the accuracy of lesion prediction reported by Landrigan et al. (30) was 76.9%, which was relatively low. In addition, only one study (22) preliminarily analyzed the consistency between the model judgment and the clinical evaluation of speech therapists, and most models have not yet verified their application effects in real clinical scenarios. Overall, in the tasks of diagnosing and classifying aphasia, the support vector machine and random forest models based on neuroimaging data performed the best, and most studies reported high accuracy rates and AUC values.

#### 3.6.2 Assess and predict the patient’s severity

Five studies (21, 23, 24, 28, 36) constructed machine learning models for predicting the severity of aphasia by integrating multi-dimensional features such as neuroimaging and language samples. The model function is mainly used to achieve objective and quantitative severity assessment, but it has some emphasis on application scenarios. One study (28) used diffusion-weighted imaging data from the acute phase to construct an early stroke prediction model, while four studies (21, 23, 24, 36) focused on fine prediction in the chronic phase. Five studies used a variety of indicators to evaluate the performance of the model, such as the F1 score of 0.70 (24), the accuracy rate of 61% (28), and the prediction performance of the model was different. In addition, these studies have mainly focused on the validation of model performance, and their application in actual clinical decision-making or improvement in patient outcomes has not been reported. Based on the existing research, the combination of neuroimaging and deep learning models (such as convolutional neural networks) demonstrates a relatively good predictive ability in severity assessment.

#### 3.6.3 Predict the language function and rehabilitation outcome of patients

Five studies (20, 26, 29, 32, 34) constructed models that were mainly used to predict language function or rehabilitation outcomes in patients. According to the prediction goals, it can be divided into two categories: three studies (26, 32, 34) predict the long-term language function outcome of patients, such as Hildesheim et al. (26) integrate clinical indicators and neuroimaging data to predict language function after stroke; two studies (20, 29) were then used to predict their response to specific rehabilitation treatments to distinguish treatment responders from non-responders. In terms of model performance, most models performed well. The accuracy of the support vector machine model constructed by Billot et al. (29) reached 92.7%. However, the accuracy of Marte et al. (20) model is 78.3%, suggesting that its performance needs to be improved. In addition, most models (20, 26, 29, 32) only completed internal validation, and only one study (34) conducted external validation in an independent clinical cohort, with an AUC of 0.91 for its updated model, which showed good discrimination ability. In terms of language function recovery and prediction of rehabilitation outcomes, the combination of multimodal data (such as neuroimaging combined with clinical characteristics) with machine learning models performs better.

#### 3.6.4 Monitor and evaluate the patient’s symptoms

The models constructed in five studies (25, 27, 31, 33, 35) were mainly used to monitor or assess symptoms in stroke patients with aphasia. Among them, three studies (27, 31, 35) automatically assessed speech intelligibility or identified emotional states of patients based on acoustic or time-frequency features of speech. For example, Mahmoud et al. (27) used time-frequency images as the input index of convolutional neural networks and achieved a recognition accuracy of 59.17% in the “health training - aphasia test” scenario, which was better than the commercial speech recognition platform. The other two studies (25, 33) used electroencephalogram indicators to evaluate the neural activity level. Močilnik et al. (25) used functional connectivity indicators to distinguish the state of patients before and after rehabilitation, and the accuracy of the model reached 89.4%, which showed good prediction performance. Wilmskoetter et al. (33) predicted naming task performance from brain electrical activity before pronunciation, and the model’s AUC ranged from 0.62 to 0.75, indicating moderate predictive ability. In the symptom monitoring task, speech and electroencephalogram signal data have certain application potential, but there are significant differences in model performance among different studies.

### 3.7 Quality assessment results

A systematic quality assessment was conducted on the 19 included studies, using the assessment tool provided by the JBI Evidence-Based Healthcare Center in Australia. Further analysis revealed that the majority of the studies had good evaluations in terms of inclusion criteria, outcome measurement, and statistical methods. However, there were still some risks of bias: small sample sizes or single-center designs might limit the generalizability of the models; a few studies did not systematically identify or adjust for potential confounding factors; some quasi-experimental studies or randomized controlled trials did not report the concealment of allocation or blinding; and some outcome measurements were limited in terms of follow-up or multiple measurements. Overall, these studies have a certain degree of methodological reliability, but there are still areas that can be improved. They provide necessary background information for the subsequent summary and comparison of model performance.

## 4 Discussion

### 4.1 Machine learning models of post-stroke aphasia mainly use supervised machine learning algorithms

In this study, 18 studies (19–29, 31–37) used supervised machine learning algorithms, and 1 study (30) used unsupervised machine learning algorithms, indicating that supervised machine learning algorithms are dominant in the current research field of post-stroke aphasia. Supervised machine learning algorithms can analyze a large amount of existing data and learn the mapping relationship between input and output, so as to build a model capable of predicting or classifying new data (38). The commonly used algorithms include decision tree, support vector machine, random forest, and neural network, but each of them has its own strengths and limitations. For instance, support vector machines perform robustly with small-sample, high-dimensional data but are sensitive to parameter selection. Random forests can effectively evaluate the importance of features, but the interpretation of the model is limited. Neural networks can automatically extract data features and reduce manual screening, but the model construction is time-consuming, and the interpretation is complex. In this study, seven studies (19–21, 24, 27, 29, 31) used a variety of algorithms to build models and compare them in order to obtain the best model, but the best algorithm recommended by different studies was different. Hu et al. (21) and Teghipco et al. (24) recommended the use of support vector regression and a convolutional neural network, respectively. This heterogeneity may be related to the function of the model, the research objective, and the characteristics of the data set. In summary, there are various types of machine learning algorithms used in post-stroke aphasia research. Future research needs to comprehensively consider algorithm characteristics, clinical problem requirements, data characteristics, and model interpretability to select and construct appropriate models. At the same time, the effectiveness of the model should be continuously evaluated and optimized through external independent validation and prospective clinical tests, so as to provide better support for individualized diagnosis and treatment and rehabilitation management for patients.

### 4.2 Application advantages of machine learning models for post-stroke aphasia

Based on the 19 included studies, machine learning models demonstrated significant advantages in post-stroke aphasia. First, the model significantly reduces the reliance on subjective experience in traditional assessments by quantifying objective biomarkers. For example, the model based on resting-state functional magnetic resonance imaging metrics can objectively distinguish aphasia and non-aphasia stroke patients, and its AUC value is 0.92 (19). Other studies identify subtle changes in patients’ emotional states by analyzing the acoustic features of speech rather than the auditory perception of therapists (35). Secondly, the model realizes the efficient analysis and automatic processing of massive and complex data, improves the evaluation efficiency, and expands the application scenarios. Language sample analysis, which is traditionally time-consuming and labor-intensive, can be used to quickly extract features and estimate aphasia quotient through machine learning (23). The accuracy of automated assessment of speech clarity can exceed 97% (31), which provides a feasible aid for low-resource Settings or remote rehabilitation. Finally, the model showed strong predictive power. The model can integrate multi-dimensional information and predict the rehabilitation potential and outcome of individuals before treatment, which provides a scientific basis for the formulation of personalized intervention programs. For example, for predicting whether a bilingual aphasia patient can benefit from a specific treatment, the F1 score ranges from 0.767 to 0.790 (20). Similarly, when predicting the potential response of a chronic aphasia patient to comprehensive rehabilitation, the F1 score reaches 0.941 (29), thus providing decision support for the development of individualized rehabilitation intervention programs.

### 4.3 Machine learning models of post-stroke aphasia have a wide range of uses, but need to be further optimized

Although machine learning models have shown wide potential for aphasia classification, severity assessment, rehabilitation prediction, and symptom monitoring, several challenges remain in their construction and application. In diagnosis and classification, most of the existing models follow the traditional clinical classification (19, 37), while attempts to independently explore new subtypes from data using unsupervised learning are not enough (30). In the future, unsupervised learning and other methods should be further used to analyze large-scale, multimodal data to discover new subtypes with more neuropathological bases or prognostic significance. In terms of severity assessment and prediction, most models rely on single-center, small-sample data (21, 23, 24, 28, 36), and their validation is mostly limited to internal cross-validation, which limits their clinical generalization ability and credibility. In the future, we should focus on building large-scale, prospective, multi-center datasets and conducting rigorous external validation. In terms of language function and rehabilitation outcome prediction, most studies remain in the performance Validation stage and lack prospective clinical utility studies (20, 26, 29, 32, 34), which fail to confirm the improvement of rehabilitation outcomes of patients. The key to the next step is to carry out prospective clinical trials covering key rehabilitation decision-making Windows such as acute and subacute phases. The existing models used to monitor symptoms are mostly based on restricted task scenarios, such as analyzing speech intelligibility (31) or predicting naming accuracy (33) through picture naming tasks, which cannot reflect the complex situation in daily natural communication. In the future, multimodal dynamic monitoring models based on natural communication scenarios should be developed to achieve more realistic and dynamic monitoring of communication ability and emotional state. Further analysis indicates that different machine learning algorithms exhibit varying performance in different tasks. Support vector machines and random forests perform robustly in small sample studies, while deep learning models (such as convolutional neural networks) show better results in severity assessment and rehabilitation outcome prediction, but they require a larger sample size and may suffer from overfitting in small sample studies. In terms of data sources, most studies were designed as single-center studies with moderate or small sample sizes, and there was insufficient external validation, which may limit the generalization ability of the models. Furthermore, the performance of the voice and EEG data fluctuates greatly in the symptom monitoring task, suggesting that the selection of data modalities has a significant impact on the model’s performance. Overall, these methodological limitations provide directions for improvement in future model optimization and clinical applications.

## 5 Summary

This review included 19 machine learning studies on post-stroke aphasia. It systematically summarized the application of algorithms, data modalities and model performance under different task types, and conducted a quality assessment by combining tools. This study not only collated the existing research results, but also evaluated the quality of the literature, sample size and validation methods, providing a reference for subsequent research. It is worth noting that there are still several major gaps in the existing research, including: the lack of external validation, the dominance of single-center and small-sample studies, insufficient integration of multimodal data, and the absence of real clinical application and prospective validation. These gaps indicate the direction for future model development and clinical implementation, and efforts should be focused on building multi-center, large-sample, and multimodal datasets, as well as conducting external validation and clinical scenario testing. Furthermore, it is necessary to explore the application of algorithm optimization and dynamic monitoring models in real clinical scenarios, in order to enhance the robustness and clinical feasibility of the models, and thereby better support individualized diagnosis and rehabilitation decisions for patients with post-stroke aphasia.
